# Clinical efficacy of hyaluronic acid in peri-implantitis treatment: a systematic review of clinical, radiographic, and biological outcomes

**DOI:** 10.2340/biid.v13.45746

**Published:** 2026-04-14

**Authors:** Raid Khayat

**Affiliations:** Oral and Maxillofacial Surgery Department, King Abdulaziz University, Jeddah, Saudi Arabia

**Keywords:** hyaluronic acid, hyaluronan, periimplantitis, dental implant, peri-implantitis

## Abstract

**Objective:**

This systematic review evaluated the impact of adjunctive hyaluronic acid (HA) on outcomes in the management of peri-implantitis, while characterizing the various protocols and formulations.

**Materials and methods:**

In accordance with the PRISMA 2020 guidelines, a search was conducted across five databases up to February 10, 2025 to identify randomized controlled trials (RCTs) and non-randomized clinical studies including controlled studies and case series. A dual-pass screening and data extraction process was employed to ensure data accuracy. The risk of bias was evaluated using Cochrane Risk of Bias 2, Risk of Bias in Non-Randomized Studies of Interventions-I, and Joanna Briggs Institute tools. The certainty of evidence for key outcomes was graded with the GRADE approach.

**Results:**

Six studies (four RCTs, one controlled pilot study with a split-mouth design, and one prospective case series) with 110 patients were included; sample sizes ranged from 5 to 63 participants, and follow-up periods ranged from 15 days to 12 months. The findings were heterogeneous; while some studies demonstrated statistically significant improvements in probing pocket depth (PPD) and bleeding on probing (BOP), other studies reported no significant difference compared to controls. Marginal bone loss (MBL) outcomes were inconsistent. HA demonstrated an anti-inflammatory effect by reducing interleukin-1 beta levels and showed some benefits against the peri-implantitis-associated bacterial community.

**Conclusion:**

Adjunctive HA is associated with favorable effects on PPD and BOP, but with a low certainty of evidence, and may have a benefit in decreasing the levels of early-colonizing bacteria. Significant improvement in MBL was associated only with surgical approaches but with a very low certainty of evidence. The high degree of heterogeneity in study design, HA formulations, and treatment protocols, coupled with a high risk of bias, makes it difficult to draw a definitive conclusion. Well-designed RCTs are required to establish the clinical role of HA in the management of peri-implantitis.

KEY MESSAGES

- Adjunctive HA appears to be effective in improving BOP and PPD with a low certainty of evidence.
- The observed benefit of HA for marginal bone gain was associated with reconstructive surgical procedures but with a very low certainty.
- Adjunctive high-molecular-weight HA showed a potent anti-inflammatory effect and had a significant impact on the bacterial profile in peri-implantitis.

## Introduction

Dental implants are susceptible to peri-implant diseases, first described as ‘périimplantose’ by Levignac in 1965 [1]. Peri-implant mucositis, in which plaque plays a central pathogenic role, is characterized primarily by bleeding on probing (BOP), with or without the presence of erythema, swelling, and suppuration (SUP).

Peri-implantitis, although a distinct disease, represents the progression of this condition. It is diagnosed by the presence of clinical signs of inflammation, increased probing pocket depth (PPD), and is marked by bone loss. The associated coronal bone loss around the intraosseous implant is defined as progression beyond expected remodeling, relative to the baseline radiographic examination at the time of loading. In the absence of baseline records, PPD ≥ 6 mm and coronal bone loss ≥ 3 mm serve as clinical diagnostics as proposed by the 2017 World Workshop [2].

The management of peri-implantitis remains a clinical challenge as standardized guidelines are not yet fully established. The decision-making varies among clinicians due to factors such as heterogeneity of bone defects and a lack of consensus on the most effective protocols [3, 4]. A primary clinical decision involves choosing between the removal of the failed/failing implant with a poor prognosis or attempting therapeutic intervention if a favorable prognosis is anticipated. Non-surgical treatment is the initial step but is generally considered insufficient; however, adjunctive antimicrobials such as metronidazole, minocycline, or chlorhexidine (CHX) may improve outcomes.

Surgical approaches are required due to restricted instrumental access or in cases where inflammatory signs are pronounced. These include access flap, debridement, and local delivery of drugs. Moreover, resection of the pathological pockets and regeneration of bony defects may also be considered [5].

The exploration of hyaluronic acid (HA) as a potential therapeutic candidate is driven by its promising results and reported benefits in periodontal tissues where the biological mechanisms of inflammation and tissue repair closely mirror those of the peri-implant environment. HA has been shown to promote the proliferative and migratory functions of fibroblasts, in addition to a strong influence on osteoprogenitor cell growth using formulations marketed for tissue regeneration. Furthermore, structural modifications of HA, such as cross-linking, slow down its degradation and increase its rheological stability at the injury site. These advantages for both soft and hard tissues, namely wound healing and bone regeneration, may produce positive outcomes in reconstructive surgeries [6, 7].

Animal studies further demonstrated the regenerative potential of HA, with histological evidence of new bone, cementum, and connective tissue attachment [8, 9]. Furthermore, clinical studies have shown benefits in soft tissue repair [10, 11], in treating moderate to severe chronic periodontitis [12], and in improving clinical parameters in suprabony defects [13]. Additionally, microbial reduction was reported in both smokers and non-smokers with chronic periodontitis [14]. Both periodontal and peri-implant diseases share a primary etiology of plaque-associated biofilm; moreover, periodontitis is a recognized risk factor for peri-implantitis [2, 5], and HA may offer a similar antimicrobial effect in the management of peri-implantitis.

Given these positive findings, investigating HA’s clinical potential in the peri-implant context is a logical progression. Thus, the objective of this systematic review was to synthesize and critically evaluate the clinical evidence regarding the efficacy of HA in the management of peri-implantitis.

## Materials and methods

### Review protocol

This systematic review was conducted and reported in accordance with the PRISMA 2020 statement; a completed PRISMA 2020 checklist is provided as Supplementary Table 1. A PRISMA flow diagram was used to document the study selection process. The protocol for this systematic review was registered with the International Prospective Register of Systematic Reviews (PROSPERO) (Registration ID: CRD420251007717).

### Eligibility criteria

The inclusion criteria were based on the PICO framework.

**Problem (P):** Patients diagnosed with peri-implantitis.

**Intervention (I):** Treatment involving the use of HA as an adjunct to non-surgical or surgical therapy.

**Control (C):** Placebo, no additional treatment, or active comparators.

**Outcome (O):** Changes in clinical parameters (PPD, BOP, SUP, clinical attachment level, radiographic bone level change) and other relevant outcomes (e.g. biomarkers, adverse events).

### Inclusion criteria

English-language publications of randomized controlled trials (RCTs) and non-randomized clinical studies, including controlled studies and case series, that utilized HA in the treatment of peri-implantitis were included without a restricted time frame on publication dates.

### Exclusion criteria

- Studies reporting the use of HA as a preventative measure for peri-implantitis or for the maintenance of dental implants
- Studies reporting on the treatment of peri-implant mucositis or deficient soft tissue only
- Studies that did not utilize HA in the treatment of peri-implantitis
- Studies conducted *in vitro* or on animals
- Reviews, case reports, editorials, and grey literature (e.g. conference abstracts and trial registries)

### Search strategy and terms

The search was initially conducted in September 2024 and repeated on February 10, 2025, across the following databases: PubMed, Cochrane Central Register of Controlled Trials, Embase, Web of Science, and Scopus. A combination of the following main search terms was used: peri-implantitis and HA. The exact search strategy was tailored for each database to optimize the results, with the core terms being ((‘Hyaluronic Acid’ OR ‘Hyaluronan’) AND (‘Peri-implantitis’ OR ‘Peri-implant’ OR ‘Dental implant’)). Full search strategy is presented in Supplementary Table 2.

### Study selection and data extraction

The titles and abstracts of the identified studies were screened by the author on the basis of the inclusion and exclusion criteria, followed by a full-text assessment of potentially relevant articles; reasons for exclusion were documented. To ensure accuracy and minimize selection bias, a dual-pass screening and extraction process was employed by the author at two distinct time points. The extracted data included study characteristics, participants’ demographics, peri-implantitis diagnosis criteria, HA specifications, details of the intervention, outcome measures, and results. Finally, the data set was reviewed and appraised by other team members to ensure accuracy.

### Risk of bias and certainty of evidence assessment

The methodological quality was assessed using different tools corresponding to each study design. The Cochrane Risk of Bias 2 (RoB 2) tool was used to assess RCTs. The non-randomized controlled pilot study with a split-mouth design was assessed using the Risk of Bias in Non-Randomized Studies of Interventions (ROBINS-I) tool. The Joanna Briggs Institute (JBI) tool was used for the prospective case series study [15]. Furthermore, the certainty of evidence for the main clinical outcomes (PPD, BOP, MBL [marginal bone loss]) was assessed using the Grading of Recommendations Assessment, Development and Evaluation (GRADE) approach and processed with the GRADEpro Guideline Development Tool (GDT) software.

## Results

**Figure 1:**
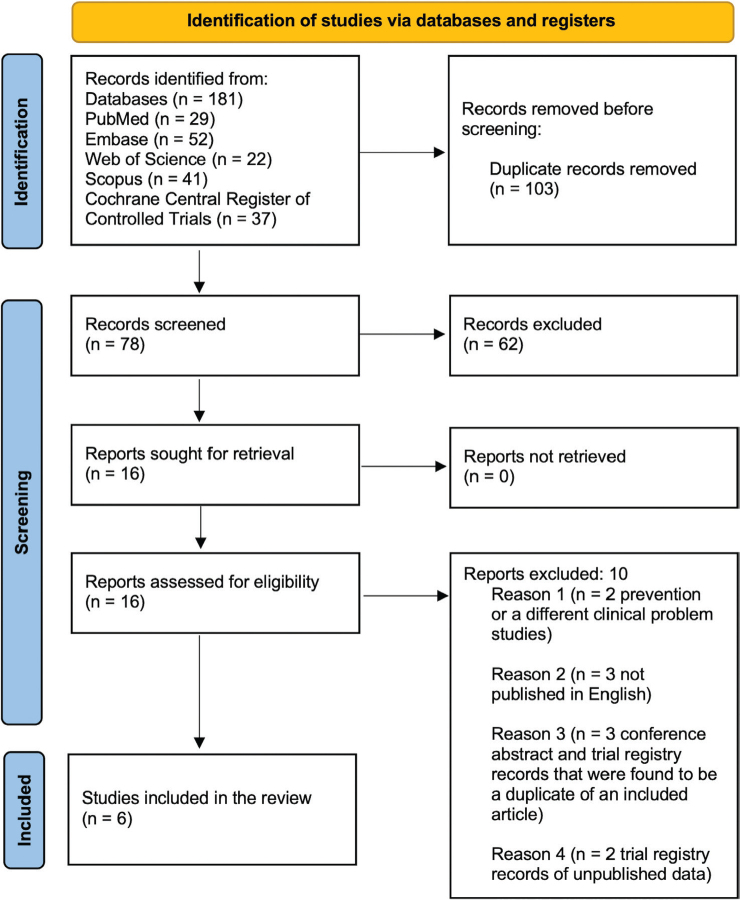
PRISMA flow diagram. This figure illustrates the flow of studies identified, screened, and included in the systematic review on HA for the treatment of peri-implantitis.

This review included six published articles: four RCTs, one controlled pilot study with a split-mouth design, and one prospective case series (Table 1). All included studies were conducted in Europe, and were published between 2009 and 2024, with a notable gap in publications meeting the inclusion criteria between 2009 and 2017. The study selection process, including the number of articles identified, screened, and ultimately included in the review, is visually summarized in the PRISMA flow diagram (Figure 1).

**Table 1:** Randomized clinical trials and non-randomized clinical studies included in the systematic review.

Study	Author/Year/Country	Journal	Study design
Hyaluronic acid reduces inflammation and crevicular fluid IL-1β concentrations in peri-implantitis: a randomized controlled clinical trial	Sánchez-Fernández et al. [16]/2021/Spain	J Periodontal Implant Sci	Randomized controlled clinical trial
Short-term effects of hyaluronic acid on the subgingival microbiome in peri-implantitis: A randomized controlled clinical trial	Soriano-Lerma et al. [17]/2020/Spain	J Periodontol	Randomized controlled clinical trial
Reconstructive peri-implantitis therapy by using bovine bone substitute with or without hyaluronic acid: a randomized clinical controlled pilot study	Rakašević et al. [18]/2023/Serbia	J Funct Biomater	Randomized controlled pilot study
Non-surgical treatment of peri-implant pockets: an exploratory study comparing 0.2% chlorhexidine and 0.8% hyaluronic acid	De Araújo Nobre et al. [19]/2009/Portugal	Can J Dent Hyg	Randomized controlled exploratory study
The use of hyaluronic acid as an adjuvant in the management of peri-implantitis	Lopez et al. [20]/2017/Italy	J Biol Regul Homeost Agents	Controlled pilot study with a split-mouth design
Reconstructive surgical therapy of peri-implant defects with ribose cross-linked collagen matrix and crosslinked hyaluronic acid: a prospective case series	Friedmann et al. [21]/2024/Germany	Clin Oral Investig	Prospective case series

### Sample characteristics

The sample sizes ranged from 5 to 63 patients (Table 2). The studies investigated the use of HA as a non-surgical or surgical adjunct for the treatment of peri-implantitis, with follow-up periods ranging from 15 days to 12 months. Two publications [16, 17] reported different outcomes from the same patient cohort within a single clinical trial registry. Their outcomes were analyzed separately without double-counting the sample size.

**Table 2:** Sample size summary.

Study	Sample size (patients)	Sample size (implants)	Age (mean ± SD)	Lost to follow up/discontinued
Sánchez-Fernández et al. [16]	61	100 (32T, 32C1, 36C2)	Test: 60 ± 9 (*n* = 21); C1: 64 ± 7 (*n* = 20); C2: 58 ±13 (*n* = 20)	Initially, 63 patients; 2 lost to follow-up
Soriano-Lerma et al. [17]	54	108 samples from 104 implants (38T, 34C1, 36C2)	Test: 60 ± 9 (*n* = 21); C1: 64 ± 6 (*n* = 21); C2: 58 ± 12 (*n* = 21)	Initially, 63 patients; for 9 patients, no DNA amplification was achieved; 2 lost to follow-up
Rakašević et al. [18]	13	19	46.85 ± 9.96	0 lost to follow up
De Araújo Nobre et al. [19]	18	18 (HA group *n* = 9, CHX group *n* = 9)	HA group: 56.2 ± 1.7; CHX group: 58.7 ± 3.1	6 patients (5 non-compliant, 1 death; unrelated) before the 1-year follow-up
Lopez et al. [20]	5	Not specified; at least one implant in different hemi-arches	Not reported	0 lost to follow up
Friedmann et al. [21]	13	15	64.3 ± 9.75	0 lost to follow up

T = test, C1 = control group 1 (placebo), C2 = control group 2 (no treatment), HA group (treated with hyaluronic acid), CHX group (treated with chlorhexidine). HA: hyaluronic acid; CHX: chlorhexidine.

### Diagnosis of peri-implantitis

The diagnosis of peri-implantitis varied across studies. Sánchez-Fernández et al. [16] and Soriano-Lerma et al. [17] applied *the Association of Dental Implantology* (ADI) criteria that included PPD ≥ 4 mm, BOP, and MBL > 2 mm. Rakašević et al. [18] applied *the 2017 World Workshop Consensus*. De Araújo Nobre et al. [19] defined the disease by PPD ≥ 5 mm, BOP, and bone loss between the coronal and middle third of the implant. Lopez et al. [20] demarcated the presence of PPD between 4 and 7 mm, spontaneous bleeding, or BOP. Finally, PPD ≥ 6 mm and radiographic peri-implant bone loss below the implant shoulder were set by Friedmann et al. [21].

### Protocols for HA

HA was used in various protocols, formulations, and delivery methods. When combined with non-surgical treatment, HA was applied as a topical gel or for intra-pocket irrigation. In surgical reconstruction studies, HA was directly applied to defects, implant surfaces, and the matrix, or supplied as pre-mixed bone substitutes. Protocol specifications and timings are detailed in Table 3.

**Table 3:** HA use specifications, post-operative instructions, and follow-up periods.

Study	Type of HA	Was the prosthesis removed?	Comparator	Protocol	Post-operative instructions	Follow up period
Sánchez- Fernández et al. [16] 2021	0.8% HMW-HA gel (crosslinked HA, 6–7 × 10⁶ Da, Ricerfarma srl) and 0.2% HA gel	No	G1: Placebo G2: No treatment	Application in the peri-implant pocket once in the clinic then 3 times per day at home for 45 days.	Home application was to be performed by massaging the gingiva around the affected area after toothbrushing, followed by avoiding eating or drinking for 20 minutes afterward.	45 and 90 days
Soriano- Lerma et al. [17] 2020	0.8% HMW-HA gel (crosslinked HA, 6–7 × 10⁶ Da, Ricerfarma srl) and 0.2% HA gel	No	G1: Placebo G2: No treatment	Application in the peri-implant pocket once in the clinic then 3 times per day at home for 45 days.	Home application was to be performed by massaging the gingiva around the affected area after toothbrushing, followed by avoiding eating or drinking for 20 minutes afterward.	45 days
Rakašević et al. [18] 2023	(Cerabone^®^ plus, Botiss Biomaterials GmbH, Berlin, Germany)	Yes, 2 weeks before the surgical intervention.	Bovine bone substitute without HA	Surgical reconstruction using a pre-mixed bovine bone substitute with HA.	Amoxicillin (500 mg, 3 times/day) or Clindamycin (600 mg, 3 times/day) for 5 days. To avoid brushing for 3 weeks and to use CHX 0.12% twice daily for 14 days. For a period of 3 months, patients received bi-weekly professional dental hygiene to the treated area.	6 months
De Araújo Nobre et al. [19] 2009	HA 0.8% gel (Gengigel^®^, Ricerfarma, Milano, Italy)	Yes, as observed in the figures within the original paper.	CHX 0.2% gel	In-office irrigation of peri-implant pockets, then brushing at home using gel.	To avoid eating, drinking, or rinsing for at least half an hour after office treatment. To use a soft toothbrush and to apply either a 0.2% CHX gel or a 0.2% HA gel (Frequency and duration were not mentioned).	1 month then 1 year
Lopez et al., [20] 2017	(IBSA Farmaceutici Italia Srl, Lodi, Italy)	Not reported	Split-mouth design- no gel	Nebulized via spray once in the clinic then twice per day at home for 15 days.	To perform two self-administrations of nebulized HA per day for 15 days on the treated side.	15 days
Friedmann et al. [21] 2024	1.4-butanediol diglycidyl ether (BDDE)-crosslinked hyaluronic acid gel (xHyA, HyaDENT BG^®^, Regedent AG, Switzerland (1.6% cross-linked hyaluronic acid, 0.2% natural hyaluronic acid)	No	None	Surgical reconstruction with ribose cross-linked collagen matrix and application of HA on the defect walls, implant surfaces, and the matrix.	To avoid mechanical oral hygiene measures for 6 weeks with the implementation of a CHX protocol. Doxycycline 200 mg, once/day for 10 days. Ibuprofen, if needed.	Weekly during the initial 6 weeks post-op Quarterly periodontal therapy Assessments at 12 weeks Outcomes measured at the 12 months

All patients received non-surgical periodontal therapy. HA: hyaluronic acid; HMW: high molecular weight; G1: group 1; G2: group 2; CHX: chlorhexidine.

### Main outcomes

The findings for the primary and secondary outcomes are synthesized in the following paragraphs and detailed in Table 4.

**Table 4:** A summary of primary and secondary outcomes.

Outcome measure	Sánchez-Fernández et al. [16]	Soriano-Lerma et al. [17]	Rakašević et al. [18]	De Araújo Nobre et al. [19]	Lopez et al. [20]	Friedmann et al. [21]
Probing pocket depth (PPD) and clinical attachment loss (CAL)	Significant reduction in test group vs. control 1 at 45 and 90 days (*p* < 0.01). CAL decreased, but the change was not statistically significant in any group.	Not applicable	Both groups improved in PPD and CAL but without statistical differences between groups.	HA and CHX groups showed significant improvements within groups at 1 month (HA: *p* = 0.031; CHX: *p* = 0.008). Attachment level had a statistically significant change in both groups (*p* < 0.05). After 1 year, overall means of PPD and attachment level were 3.3 mm and 2.6 mm, respectively. No significant difference between groups.	Slight, statistically irrelevant difference between HA and control sides at 15 days.	Significant reduction at 12 months (3.2 ± 0.66 mm) vs. baseline (7.2 ± 1.9 mm) (*p* < 0.0001). Attachment levels were noted to be similar to those reported in literature.
Bleeding on probing (BOP)	Greatest reduction in HA group at 45 and 90 days; borderline significance vs. no-product control at 90 days (*p* = 0.07).	Not applicable	Complete reduction in HA group at 6 months; control group 0.17 ± 0.39; no statistical significance.	Mean modified bleeding index scores: HA (2.2 baseline, then 1 after 1 month); CHX (1.9 baseline, then 0.7 after 1 month). After 1 year, the overall mean was 0.3.	Improvement in both HA-treated and non-HA-treated sites; no greater improvement on HA side.	Significant reduction in BOP frequency from 63% at baseline to 10% at 12 months (*p* < 0.001).
Marginal bone loss (MBL)/bone gain	Slight decrease in HA group (3.41 to 3.39 mm); increased loss in control groups; no statistical significance. Assessed via parallel periapical radiographs.	Not applicable	Significant bone gain in both groups (*p* < 0.05). Statistically greater vertical bone gain in HA group (mesial, distal, oral sites) vs. control (*p* < 0.05). Assessed via CBCT.	The post-treatment description remained similar to baseline when assessed at the 1-month follow-up. MBL not evaluated at 1-year follow up. Assessed via periapical radiographs.	Radiographic assessment of MBL was not reported.	Significant mean marginal bone gain of 1.02 ± 0.64 mm at 12 months vs. baseline (5.83 ± 2.63 mm) (*p* < 0.001). Assessed via parallel periapical radiographs. Significant defect reduction (69.1% gain of mineralized tissue).
Microbiome changes	Not reported	The HA group showed a significant decrease in several bacterial genera, including *Streptococcus*, *Veillonella*, *Rothia*, *Granulicatella*, *Prevotella*, and *Campylobacter*. Control groups (placebo and no treatment) exhibited increases in various bacterial genera such as *Propionibacterium*, *Neisseria*, *Rothia*, *Pseudomonas*, *Mycoplasma*, *Atopobium*, and *Anaeroglobulus*, while *Porphyromonas* decreased in the no-treatment control. No significant changes were observed in stratum 1 of the HA group.	Not reported	Not reported	Not reported	Not reported
Secondary outcomes	**Pro-inflammatory cytokines (IL-1β, TNF-α):** Significant reduction of IL-1β in test group compared to no treatment group (*p* = 0.04).	No secondary outcomes.	**ISQ:** Test group showed statistically significant higher scores vs. control group at 3 and 6 months (*p* = 0.009 and *p* = 0.032).	No secondary outcomes.	100% improvement in tartar and plaque indices on both sides without statistical significance.	**Defect reduction:** Newly mineralized bone area of 27.65 ± 18.50 mm² (*p* < 0.0001, 69.1% gain of mineralized tissue).
Adverse events	No adverse events were reported.	No adverse events were reported.	Two patients with flap dehiscence (healed uneventfully).	No adverse events were reported.	No adverse events were reported.	No premature interventions. Uneventful healing.

HA: Hyaluronic acid; CHX: chlorhexidine; CBCT: Cone-beam computed tomography; IL-1β: Interleukin-1β; TNF-α: tumor necrosis factor-α; ISQ: Implant stability quotient.

### PPD and clinical attachment loss

Regarding intragroup findings, a reduction in PPD within the HA groups was observed in most studies; however, the evidence for a statistically significant intergroup benefit with HA was inconsistent. One RCT reported a statistically significant PPD reduction for the HA group compared with a placebo control at 45 and 90 days (*p* < 0.01) [16]. In contrast, two other RCTs found no significant difference in PPD between the HA and control groups, although PPD in both groups improved over time [18, 19]. The prospective case series also reported a significant PPD reduction from baseline, but this was an intragroup analysis without a control [21]. In the split-mouth study, there was a lack of numerical data and results were descriptive [20]. They reported a non-significant difference in PPD between the HA and control sides at 15 days post-treatment.

For clinical attachment loss (CAL), no study found a statistically significant difference between groups. Notably, the prospective case series reported a statistically significant increase in buccal soft tissue dehiscence (*p* < 0.0001) [21].

### Bleeding on probing

Reductions in BOP were observed in intragroup analysis; however, statistically significant intergroup differences favoring HA were not consistently observed. One RCT reported a borderline significant reduction in BOP for the HA group compared to a no-product control (*p* = 0.07) [16]. Another RCT found a complete resolution of BOP in the HA group at 6 months without statistical significance [18]. Further findings from another RCT and the split-mouth pilot study found that improvements in the HA groups were not statistically different from those seen in their respective control groups [19, 20]. In contrast, the prospective case series observed a significant reduction in BOP frequency from 63 to 10% (*p* < 0.001) [21].

### MBL/bone gain

In studies using a non-surgical approach, topical HA did not lead to bone gain. One RCT using parallel periapical radiographs reported that MBL showed a minor numerical improvement in the HA group up to a 90-day follow-up, with no significant difference compared to controls [16]. Another study also utilizing periapical imaging reported no changes from baseline after a 1-month follow-up [19].

In contrast, studies that used HA as an adjunct to surgical reconstruction reported statistically significant bone gain. One RCT found a significantly greater vertical bone gain in the HA group compared to the control group at 6 months when assessed with cone-beam computed tomography (*p* < 0.01) [18]. The prospective case series using parallel periapical radiographs reported a significant marginal bone gain at the 12-month follow-up (*p* < 0.001) [21]. The split-mouth pilot study did not report on MBL [20].

### Microbiome changes

A single RCT evaluated the effect of high-molecular-weight hyaluronic acid (HMW-HA) on the subgingival microbiome, using 16S rRNA gene sequencing to identify bacteria from peri-implantitis sites [17]. A total of 27 phyla and 604 genera were detected; 53 genera that had a higher relative abundance (> 0.1%) were analyzed and categorized into three groups (strata).

HMW-HA did not have an effect on the stratum 1, which consisted of environmental bacteria from exogenous sources, such as water, including *Ralstonia* and *Sphingomonas*. The decrease was significant (*p* < 0.05) in stratum 2, described as the early colonizers, including *Streptococcus*, *Veillonella*, *Rothia*, and *Granulicatella*, which are part of the plaque biofilm. In stratum 3, representing middle and late colonizers, a significant reduction (*p* < 0.05) was found in *Prevotella* and *Campylobacter* only; these have a strong link to periodontitis and peri-implantitis. Additionally, HMW-HA was found to lower the microbial alpha diversity within this stratum (*p* < 0.05).

### Additional outcomes

The assessment of HA’s impact on inflammatory markers is limited to a single trial, which found that topical HMW-HA significantly reduced interleukin-1β (IL-1β) levels in peri-implantitis patients with PPD ≥ 5 mm compared to the no-treatment control (*p* = 0.04) [16]. In another RCT, the HA group demonstrated statistically significantly higher implant stability quotient (ISQ) scores at both 3- and 6-month follow-ups [18]. The prospective case series reported a significant radiographic gain of 69.1% in mineralized tissue within the defect area (*p* < 0.0001) [21]. Additionally, the split-mouth pilot study reported a 100% resolution in tartar and plaque indices on both sides without statistical significance [20].

### Treatment success criteria

The criteria for treatment success varied across the studies. In a study by Rakašević et al. [18], success was defined as the absence of SUP/BOP, a PPD < 5 mm, and no further bone loss. This was achieved in 75% of the patients and 83% of the implants in both study groups.

De Araújo Nobre et al. [19] defined success as the absence of SUP/BOP, a PPD ≤ 4 mm, attachment level improvement, and the absence of mobility. They reported the absence of SUP for all implants post-operatively. The HA group had a 55% success rate, whereas the CHX group had an 89% success rate; however, this difference was not statistically significant. They suggested that HA is more suitable for cases with a PPD ≤ 5 mm, having observed only one successful outcome in a 6 mm pocket in their sample. Additionally, the prospective case series protocol resulted in healthy, non-inflamed peri-implant conditions and consistently resolved the intrabony defects [21].

### Risk of bias assessment

Three RCTs were found to have some concerns, mainly due to retrospective registration of the trial protocol, missing data, and an unblinded (no treatment) group (Table 5). One RCT was found to have a high risk of bias due to lack of a pre-registered protocol, lack of allocation concealment, missing data, and unblinding of both the patients and assessors.

**Table 5:** Risk of bias for the four included RCTs using the domains of the Cochrane RoB 2 tool.

Author (Year)	RoB 2 domain 1 (Randomization)	RoB 2 domain 2 (Interventions)	RoB 2 domain 3 (Missing data)	RoB 2 domain 4 (Outcome measurement)	RoB 2 domain 5 (Reported result)	Overall risk of bias
Sánchez-Fernández et al. [16] (2021)	Low	Some concerns (Unblinded group 2)	Some concerns (Missing data)	Low	Some concerns (Retrospective registration of trial protocol)	**Some concerns**
Soriano-Lerma et al. [17] (2020)	Low	Some concerns (Unblinded group 2)	Some concerns (Missing data)	Low	Some concerns (Retrospective registration of trial protocol)	**Some concerns**
Rakašević et al. [18] (2023)	Low	Low	Low	Low	Some concerns (Retrospective registration of trial protocol)	**Some concerns**
De Araújo Nobre et al. [19] (2009)	Some concerns (Lack of allocation concealment)	High (Participants were unblinded) Absence of standardized self-care protocol	High (Missing outcome data)	High (Unblinded outcome assessors)	High (Lack of a pre-specified analysis plan, protocol not registered)	**High**

RoB 2: Risk of Bias 2; RCTs: randomized controlled trials.

The controlled pilot study with a split-mouth design was found to have a critical risk of bias (Table 6). This judgment was based on lack of randomization, the small sample size, deviations from intended interventions, unblinding of examiners, substantial amount of missing data, and absence of a pre-registered protocol. The prospective case series was well conducted and demonstrated methodological strengths. However, it was determined to have a high risk of bias due to the inherent limitation of lacking a control group (Table 7).

**Table 6:** Risk of bias for the controlled pilot study with a split-mouth design using the ROBINS-I.

Author (Year)	Domain-specific judgments/justification	Overall risk of bias
Lopez et al. [20] (2017)	• **D1 (Confounding):** Serious risk (No randomization) • **D2 (Selection):** Critical risk (Small sample size, non-random sample with unclear selection process) • **D3 (Intervention):** Moderate risk (Concerns about validity of home treatment delivery method) • **D4 (Deviations):** Moderate risk (Adherence to self-treatment not monitored) • **D5 (Missing data):** Low risk (No reported patient dropouts) • **D6 (Measurement):** Serious risk (No blinding of examiners) • **D7 (Reported result):** Serious risk (No pre-registered protocol and lack of numerical/statistical data)	**Critical risk**

D: Domain.

**Table 7:** Risk of bias for the prospective case series using the JBI checklist.

Author (Year)	JBI checklist
Friedmann et al. [21] (2024)	Were there clear criteria for inclusion in the case series? **Yes** Was the condition measured in a standard, reliable way for all participants included in the case series? **Yes** Were valid methods used for identification of the condition for all participants included in the case series? **Yes** Did the case series have consecutive inclusion of participants? **Unclear** Did the case series have complete inclusion of participants? **Unclear** Was there clear reporting of the demographics of the participants in the study? **Yes** Was there clear reporting of clinical information of the participants? **Yes** Were the outcomes or follow up results of cases clearly reported? **Yes** Was there clear reporting of the presenting site(s)/clinic(s) demographic information? **No** Was statistical analysis appropriate? **Yes** Overall appraisal: **Include**

JBI: Joanna Briggs Institute.

### Certainty of evidence

The initial certainty for both PPD and BOP was rated as high due to the inclusion of RCTs; however, it was downgraded to low certainty because of the risk of bias and imprecision. Similarly, the initial certainty for MBL was high but was downgraded to very low certainty because of the risk of bias, imprecision, and inconsistency of the findings. Detailed explanations are presented in Table 8.

**Table 8:** GRADE certainty of evidence summary for PPD, BOP, and MBL.

Certainty assessment							Certainty	Importance
No of studies	Study design	Risk of bias	Inconsistency	Indirectness	Imprecision	Other considerations		
**Probing pocket depth (assessed with: Periodontal probe)**								
5	Randomized trials and non-randomized studies	Serious^a^	Not serious	Not serious	Serious^b^	Publication bias: No evidence was found, but not formally assessed	⨁⨁◯◯ Low^a,b^	Critical
**Bleeding on probing (assessed with: Periodontal probe)**								
5	Randomized trials and non-randomized studies	Serious^a^	Not serious	Not serious	Serious^b^	Publication bias: No evidence was found, but not formally assessed	⨁⨁◯◯ Low^a,b^	Critical
**Marginal bone loss (assessed with: Radiographs)**								
4	Randomized trials and non-randomized studies	Serious^c^	Serious^d^	Not serious	Serious^e^	Publication bias: No evidence was found, but not formally assessed	⨁◯◯◯ Very low^c,d,e^	Critical

PPD: probing pocket depth; BOP: bleeding on probing; MBL: marginal bone loss; RCTs: randomized controlled trials.

Explanations

a Downgraded one level for serious risk of bias: Evidence base includes two RCTs with some concerns, one RCT with high risk, one controlled split-mouth study with critical risk, and one prospective case series.

b Downgraded one level for serious imprecision: Small sample size of patients receiving the intervention (*n* = 110).

c Downgraded one level for serious risk of bias: Evidence base includes two RCTs with some concerns, one RCT with high risk, and one prospective case series.

d Downgraded one level for serious inconsistency: Results were highly variable across studies; findings ranged from slight insignificant gain, significant gain, or bone stability.

e Downgraded one level for serious imprecision: Small sample size of patients receiving the intervention (*n* = 105). The sample size is lower than for clinical outcomes (*n* = 110) because Lopez et al. [20] did not report MBL.

## Discussion

### Clinical and radiographic outcomes

This systematic review found that while adjunctive HA was associated with improvements in clinical parameters such as PPD and BOP, the evidence for its superiority over control treatments was limited and of low certainty. Furthermore, findings regarding the optimal PPD for HA treatment are conflicting around the 5 mm threshold. One study observed a strong anti-inflammatory effect in deeper pockets [16], while another suggested that HA success is restricted to shallower sites [19]. Furthermore, a clear benefit for CAL with the use of HA could not be established from the included studies.

Marginal bone findings were highly dependent on the treatment modality. Notably, a significant benefit for MBL was observed only when HA was used as part of a reconstructive surgical protocol [18, 21]. In contrast, the two studies that utilized the non-surgical approach showed no significant changes in MBL [16, 19]. The intervention approach appears to contribute directly to this heterogeneity in outcomes. This suggests that the efficacy of HA in treating bone loss may be highly dependent on surgical intervention, which appears necessary for achieving a therapeutic effect with many of the currently available HA products for intraoral use.

Cerabone^®^ plus, used in Rakašević et al. [18], is a bovine bone substitute pre-mixed with HA. A combination of multiple treatment modalities was applied in this study, including titanium brushes, photodynamic therapy, implantoplasty of exposed threads, post-operative antibiotics, and CHX use. Such multimodal protocols make isolating the true effects of HA particularly challenging.

In the prospective case series by Friedmann et al. [21], HyaDENT BG^®^ was applied to the implant and defect wall surfaces, as well as the ribose cross-linked collagen matrix, to augment the defects without the addition of bone substitutes. Despite the positive bone gain outcome, the reconstructive treatment resulted in a statistically significant soft tissue dehiscence. This recession should be viewed as a potential consequence of the overall treatment approach and the pre-operative reported stage of bone loss rather than a specific result of the HA component.

In the included non-surgical treatment studies, there was stability in MBL, but no evidence of a statistically significant bone gain with topical HA. Both Sánchez-Fernández et al. [16] and De Araújo Nobre et al. [19] assessed their findings using periapical radiographs. However, the former study reported MBL in millimeters up to a 90-day follow-up, whereas the latter’s description of MBL was less accurate, as it would not detect small changes by reporting its level according to the coronal, middle, or apical thirds of the implant. This was assessed after a short follow-up of only 1 month, which may be insufficient to detect radiographic changes.

Despite being a crucial component in peri-implantitis assessment, radiographic data were not evaluated in Lopez et al. [20]. Furthermore, the radiographic assessment using 2D and 3D imaging techniques may introduce variations in how subtle volumetric bone changes are captured. Given the limited body of evidence, the high variation in HA manufacturing, and clinical protocols, it remains challenging to definitively establish the efficacy of HA in facilitating marginal bone gain or reliably stabilizing MBL to arrest disease progression.

### Biological and microbiological effects

IL-1 and tumor necrosis factor (TNF) are key markers of destructive periodontal disease, as they stimulate a cascade of events that amplify the inflammatory response, leading to bone resorption and attachment loss [22]. Both IL-1β and TNF levels are found to be elevated in the gingival crevicular fluid of patients with chronic periodontitis compared to healthy individuals, with particularly high levels in older patients. Suppressing these factors may mitigate the severity of the disease by reducing inflammation and osteoclastic activity. In this regard, non-surgical periodontal treatment has been shown to significantly lower IL-1β levels [23].

The observed reduction of IL-1β levels in the included RCT is consistent with the broader understanding that HMW-HA has anti-inflammatory effects, whereas low-molecular-weight HA is pro-inflammatory [6, 16]. The findings of Soriano-Lerma et al. [17] suggest that peri-implantitis can be caused by either opportunistic non-oral bacteria or common oral bacteria. The use of HMW-HA in early stages of peri-implantitis may disrupt the bridging towards biofilm maturation with late-stage bacterial colonies.

### Comparison with existing literature

Non-surgical treatment of peri-implantitis is expected to improve clinical outcomes by 20–50% in BOP and achieve a ≤ 1 mm reduction in PPD; however, it is unlikely to resolve advanced cases [24]. In a randomized clinical trial that assessed the efficacy of adjunctive systemic antibiotics in the treatment of peri-implantitis, all patients were treated with non-surgical therapy and instructed to use CHX twice a day for 4 weeks. The test group received a combined amoxicillin and metronidazole regimen. However, this did not have a statistical significance in reducing PPD as the primary measured outcome. Moreover, both groups did not show improvement in BOP [25].

In a review evaluating bone regeneration in peri-implantitis treatments, it was reported that vertical bone gain is usually limited and not as predictable as the improvement in clinical signs including BOP and PPD. In addition, the amount of bone fill is highly dependent on the defect morphology, with four-wall defects having a better prognosis. Moreover, initial deeper vertical defects were found to have a significantly greater radiographic defect fill after 1 year of follow-up compared to shallower ones [26].

In a systematic review and meta-analysis assessing peri-implant tissue changes, reconstructive surgery was associated with better outcomes when compared to non-reconstructive surgeries. They reported a mean bone gain of 1.95 mm (95% CI [0.05; 3.86], *p* = 0.044) and a reduction of PPD by 1.27 mm (95% CI [−1.96; −0.60], *p* < 0.001) [27]. Another systematic review and meta-analysis assessing peri-implant osseous defects, found similar positive results for bone gain. Reconstructive surgical techniques in the treatment of peri-implantitis had better outcomes than open flap debridement in terms of bone refill with a mean gain of 1.01 mm (95% CI: 0.55–1.46; *p* = 0.0001). However, no significant differences were found between the approaches in improving BOP and PPD [28].

Many findings of the study by Soriano-Lerma et al. [17] are supported by a meta-analysis of the microbiota associated with peri-implantitis, which associates the disease with specific pathogens, including *Porphyromonas gingivalis*, *Tannerella forsythia*, *Treponema denticola*, *Fusobacterium nucleatum*, and *Prevotella intermedia* [29], all of which were found to fall within the third stratum. Furthermore, viruses such as human herpesvirus 4, Epstein–Barr 1, and cytomegalovirus 2 were detected in peri-implantitis sites [30]. Still, a single, universal microbial profile for peri-implantitis has yet to be agreed upon, but in essence there is general consensus that peri-implantitis sites are not uniform in their bacterial composition [31].

The literature reports no strong evidence to suggest the most effective treatment intervention for peri-implantitis. The adjunctive use of CHX in non-surgical peri-implantitis therapy was not found to have a significant improvement in key clinical parameters, including PPD, BOP, and CAL; consequently, its efficacy is considered limited [32–34].

In a comprehensive overview of systematic reviews on peri-implantitis, the disease was found to likely occur after 5 years of loading the implant. Patients with a history of periodontitis, uncontrolled diabetes, cardiovascular disease, or smoking are at greater risk of developing the condition. Combining surgical and non-surgical treatments can lead to successful outcomes without identifying a single most effective one. Additionally, any non-surgical treatment combined with another intervention is more effective than debridement alone [30]. Furthermore, accumulating evidence supports the inclusion of HA in broader treatment protocols, including preventative or maintenance measures, as it has shown positive clinical outcomes in peri-implant health and acceleration of healing [35] and has been suggested to exert a protective action against bacterial colonization [17].

The findings presented in this review are broadly consistent with a recent systematic review and meta-analysis by López-Valverde et al. [36] and a scoping review by Bokor et al. [37], both of which conclude that HA is a promising but not yet definitively proven therapy.

However, this review is distinguished by its formal GRADE assessment, its use of specific risk of bias tools for each study design, and its in-depth critique of confounding factors within each primary study. The narrative synthesis allowed for the inclusion of a broader range of studies, providing a more comprehensive evaluation of the current literature. Furthermore, the decision that a meta-analysis was not suitable due to high heterogeneity represents a more cautious interpretation of the existing evidence, a point underscored by the substantial inconsistency (*I*^2^ up to 97%) reported in the meta-analysis by López-Valverde et al. [36].

A final key methodological difference lies in the handling of an overlapping patient cohort in two publications [16, 17]; this review identified the overlap and counted the cohort only once, using the final analyzed sample size of 61 patients. In contrast, the meta-analysis appears to have double-counted this cohort by including both studies as separate entries in their statistical pooling, using the initial enrollment number of 63 patients for each [36]. Consequently, this methodology may lead to an overestimation of the evidence’s weight and precision in a pooled statistical result.

### Limitations

Among the included studies, there was variability in the diagnostic and success criteria, with inconsistent thresholds for PPD and MBL. In addition, SUP is a recognized clinical sign of peri-implantitis; however, this measure was not consistently reported or quantified as a finding across the included studies. The lack of standardization complicates direct comparison. Furthermore, the identified methodological issues through the risk of bias assessment reduce the overall confidence in the outcomes, requiring a careful interpretation of the findings.

There was significant heterogeneity across the studies’ designs, particularly the highly variable use of HA. This was evident in the differences in its specific formulation, delivery methods, and directions for use. This lack of standardization is a key difference from agents like CHX, which have established clinical regimens and are, in most cases, prescribed by clinicians in a more uniform manner.

The studies also varied in the types of controls, follow-up durations, and the implementation of surgical reconstruction. Due to this wide variation and the limited number of studies, it is challenging to draw firm conclusions and provide clear clinical directions. Therefore, a meta-analysis was not performed as it would have resulted in an unreliable and underpowered summary.

The small sample size of the included studies is a limitation of the precision and certainty of the evidence presented in this review. It is important to recognize that this is an expected finding, given the complexity of treating peri-implantitis, especially with the use of HA as a relatively rare and novel intervention.

A geographical limitation of this review is that all included English-language publications originated within Europe. However, this does not preclude the existence of studies published in other languages globally. Moreover, the HA formulations utilized in the studies were all produced by European-based manufacturers, which likely reflects the concentration of research in this region.

### Future research

To establish HA’s efficacy, future research should focus on standardization and well-designed clinical trials. Standardized protocols, long-term follow-up, and larger sample sizes are necessary to provide definitive evidence. Studies should also explore whether the concentration of HA influences clinical outcomes.

## Conclusion

Taking into account the high heterogeneity in protocols and HA formulations, along with significant limitations such as small sample sizes and a high risk of bias, the current evidence is insufficient to demonstrate clinical superiority over other adjunctive treatments.

Adjunctive HA contributes to improvements in BOP and PPD (low certainty); however, evidence of significant marginal bone gain was observed exclusively in conjunction with reconstructive surgical procedures whether applied with or without bone substitutes (very low certainty).

In addition to being well-tolerated, HMW-HA was found to be beneficial in decreasing the levels of early colonizer bacteria and exhibiting a potent anti-inflammatory effect. Consequently, future research should establish standard clinical protocols with attention to formulations of HA.

## Geolocation information

The clinical data included in this systematic review originated from several European countries, namely Spain, Serbia, Portugal, Germany, and Italy.

## Supplementary Material



## Data Availability

All data and materials are fully available within the published literature cited in this review and its Supplementary files.
